# Novel Key Ingredients in Urinary Tract Health—The Role of D-mannose, Chondroitin Sulphate, Hyaluronic Acid, and *N*-acetylcysteine in Urinary Tract Infections (Uroial PLUS^®^)

**DOI:** 10.3390/nu15163573

**Published:** 2023-08-14

**Authors:** Felice Crocetto, Raffaele Balsamo, Ugo Amicuzi, Luigi De Luca, Alfonso Falcone, Benito Fabio Mirto, Gaetano Giampaglia, Gianpiero Ferretti, Federico Capone, Fabio Machiella, Domenico Varriale, Enrico Sicignano, Giovanni Pagano, Alessandro Lombardi, Giuseppe Lucarelli, Francesco Lasorsa, Gian Maria Busetto, Francesco Del Giudice, Matteo Ferro, Ciro Imbimbo, Biagio Barone

**Affiliations:** 1 Department of Neurosciences and Reproductive Sciences and Odontostomatology, University of Naples Federico II, 80131 Naples, Italy; felice.crocetto@unina.it (F.C.); alfonso.falcone01@gmail.com (A.F.); fmirto22@gmail.com (B.F.M.); gaetanogiampaglia@hotmail.it (G.G.); gianpi17@hotmail.it (G.F.); fedecapone@outlook.it (F.C.); f.machiella@gmail.com (F.M.); domenicov93@libero.it (D.V.); enrisici90@gmail.com (E.S.); giovanni.pagano1@outlook.it (G.P.); alessandrolombardi1996@gmail.com (A.L.); ciro.imbimbo@unina.it (C.I.); 2 Urology Unit, AORN Ospedali dei Colli, Monaldi Hospital, 80131 Naples, Italy; raffaelebalsamo5@gmail.com; 3 Division of Urology, Department of Surgical Sciences, AORN Sant’Anna e San Sebastiano, 81100 Caserta, Italy; u.amicuzi@gmail.com; 4 Division of Urology, Department of Surgical Multispecialty, AORN Antonio Cardarelli, 80131 Naples, Italy; luigideluca86@gmail.com; 5 Urology, Andrology and Kidney Transplantation Unit, Department of Emergency and Organ Transplantation, University of Bari, 70124 Bari, Italy; giuseppe.lucarelli@inwind.it (G.L.); francesco-lasorsa96@libero.it (F.L.); 6 Department of Urology and Organ Transplantation, University of Foggia, 71121 Foggia, Italy; gianmaria.busetto@unifg.it; 7 Department of Maternal Infant and Urologic Sciences, Policlinico Umberto I Hospital, Sapienza University of Rome, 00161 Rome, Italy; francesco.delgiudice@uniroma1.it; 8 Department of Urology, IEO—European Institute of Oncology, IRCCS—Istituto di Ricovero e Cura a Carattere Scientifico, 20141 Milan, Italy; matteo.ferro@ieo.it

**Keywords:** urinary tract infection, D-mannose, chondroitin sulphate, hyaluronic acid, *N*-acetylcysteine, urothelial barrier

## Abstract

Urinary tract infections represent a common and significant health concern worldwide. The high rate of recurrence and the increasing antibiotic resistance of uropathogens are further worsening the current scenario. Nevertheless, novel key ingredients such as D-mannose, chondroitin sulphate, hyaluronic acid, and *N*-acetylcysteine could represent an important alternative or adjuvant to the prevention and treatment strategies of urinary tract infections. Several studies have indeed evaluated the efficacy and the potential use of these compounds in urinary tract health. In this review, we aimed to summarize the characteristics, the role, and the application of the previously reported compounds, alone and in combination, in urinary tract health, focusing on their potential role in urinary tract infections.

## 1. Introduction

Urinary tract infections (UTIs) are a common and significant health concern worldwide, affecting over 150 million individuals each year [1]. While both men and women can develop UTIs, with a prevalence which increases with age, the condition is more prevalent in women due to anatomical and behavioral factors such as a shorter urethra, which provides easier access for bacteria to reach the bladder, and the tendency to delay micturition. Specifically, a spike in the prevalence of UTIs is associated with young women aged 14–24 years old, while the prevalence in women over 65 years of age is approximately 20% versus 11% of the overall population [2]. UTIs occur when pathogenic microorganisms, primarily bacteria, invade the urinary tract, leading to inflammation and infection. UTIs can affect different parts of the urinary tract, including the urethra, bladder, ureters, and kidneys, albeit the most common type of UTI is a lower urinary tract infection, which primarily involves the bladder, provoking cystitis. Nevertheless, if left untreated or if the infection spreads, it can progress to an upper urinary tract infection, affecting the kidneys and thus developing pyelonephritis. UTIs can cause various symptoms, including frequent and painful urination, a persistent urge to urinate, cloudy or bloody urine, and lower abdominal discomfort. As previously described, the reason for an increased prevalence of UTIs in women is mostly related to anatomical and hormonal reasons [3]. In addition to the short length of the female urethra, which could easily allow the migration of bacteria from the anogenital area to the urethra and, therefore, to the bladder, other risk factors leading to the insurgence of UTIs are sexual activity as well as menopause [4,5]. In the first case, sexual intercourse could alter bacterial flora and vaginal pH, which, added to a lack of postcoital urination, vaginal douches and poor hygiene of the male partner could increase the risk of UTIs. In the second case, postmenopausal women report, among other common risk factors (i.e., delayed micturition, low fluid intake, and even genetic predisposition), vulvovaginal atrophy as a main causative factor which is associated with the decreased colonization of *Lactobacilli* [6]. Albeit the primary causative agent of UTIs is the bacterium *Escherichia coli*, which normally resides in the intestinal tract but can migrate to the urinary tract and cause infection, other bacteria, such as *Klebsiella pneumoniae*, *Proteus mirabilis*, and *Staphylococcus saprophyticus*, can also contribute to UTIs. In some cases, UTIs can be caused by fungal or viral pathogens, although they are less common [7,8]. In addition to previously described risk factors, urinary catheterization, urinary tract abnormalities, compromised immune function, hormonal changes, and poor hygiene practices could increase the risk of UTIs while certain populations, such as pregnant women, individuals with diabetes and the elderly, are also at higher risk of developing UTIs [6,9].

The consequences of UTIs can range from discomfort and inconvenience to more severe complications. If left untreated, UTIs can lead to recurrent infections, kidney damage, and potentially life-threatening bloodstream infections. The impact of UTIs extends beyond individual health, as they contribute to a significant economic burden through healthcare costs and productivity losses. It has been indeed estimated, in the United States, that UTIs represent over 7 million office visits and 1 million emergency department visits for a total of over 100,000 hospitalizations and an annual cost of USD 1.6 billion [10]. Commonly, the conventional treatment for UTIs involves antibiotics, which are prescribed based on the identified pathogen and its susceptibility to specific antibiotics. However, the overuse and misuse of antibiotics have led to the emergence of antibiotic-resistant bacteria, making the treatment of UTIs more challenging. As a result, there is growing interest in alternative approaches, including natural compounds and preventive strategies, to support urinary tract health and reduce the incidence of UTIs [11].

**Figure 1:**
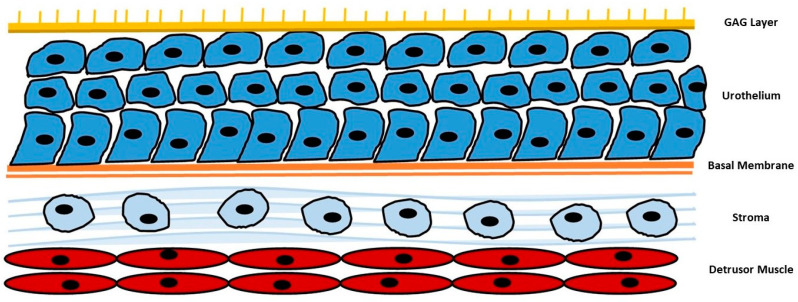
Summarization of urothelial barrier. GAG: glycosaminoglycans.

In this paper, we will focus on highlighting the potential benefits of specific natural compounds, including D-mannose, chondroitin sulphate, *N*-acetylcysteine (NAC), and hyaluronic acid, in promoting urinary tract health and preventing UTIs via the action of the urothelial barrier (Figure 1). By understanding the underlying mechanisms and evidence supporting these natural compounds, we aim to contribute to the development of effective strategies for maintaining urinary tract health and reducing the burden of UTIs.

## 2. D-mannose

**Figure 2:**
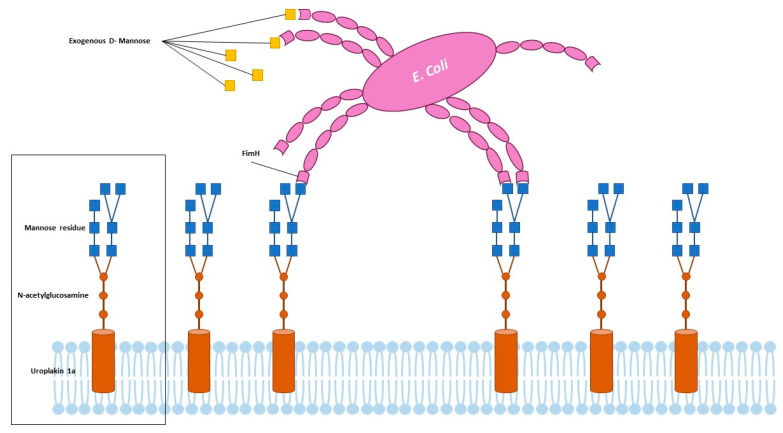
Adherence of uropathogens and role of D-Mannose.

D-mannose is a monosaccharide which is naturally found in several plants, fruits, and berries; albeit, it is also synthesized in the human body from glucose, where it contributes to glycoprotein synthesis and the glycosylation of proteins. The interest towards this compound in the treatment of UTIs dates back to the 1970s when the first episodes of the emergence of antibiotic-resistant uropathogens prompted the use of alternative methods, when feasible, to antibiotic treatment [12,13]. D-mannose has demonstrated in vitro no effects on uropathogens metabolism or growth nor interfere with antibiotic activity while, instead, could inhibit the adherence of *E. coli* to urothelial cells occupying the FimH fimbriae of the bacteria. The adhesion of pathogens to the urothelial cells prevents, indeed, their removal or washing off and represents the first step for urinary bladder colonization. The FimH fimbriae of *E. coli* interact with peptides and glycosylated residues on urothelial cells and extracellular matrix, permitting the bacteria to bind onto bladder epithelial cells. The attachments are made at the level of uroplakin 1a, which is a glycosylated protein composed of mannose molecules in the terminal units. Exogenously delivered D-mannose competitively inhibits the adhesion of bacteria to this terminal part of uroplakin 1a, saturating FimH adhesins [14,15,16] (Figure 2). Several in vivo studies have investigated the effects of D-mannose in the treatment and prevention of UTIs. Domenici et al., reported a lower recurrence rate of uncomplicated UTIs in 43 women, in the absence of side effects [17]. Similarly, Kranjcec et al. reported a significantly decreased risk of recurrent UTIs in 308 women treated with D-mannose or nitrofurantoin daily. In particular, the efficacy in decreasing the recurrent episodes of UTIs was similar among the groups treated with nitrofurantoin or D-mannose [18]. The efficacy of D-mannose was also reported in men, as described by Palleschi et al., which used a combination of NAC and D-mannose, compared to prulifloxacin, in patients undergoing urodynamic examinations. The association of D-mannose and NAC yielded similar results compared to antibiotics, in preventing UTIs in patients undergoing urodynamic examination [19]. Additionally, the use of D-mannose permitted a decrease in the perception of lower urinary tract symptoms as well as the incidence of UTIs in women undergoing prolapse surgery, as reported by Russo et al., in a recent study [20]. Analogous results were obtained in other studies utilizing D-mannose alone or in combination with other nutraceutical products or therapies [21,22,23,24]. Lastly, Lenger et al., published, in 2020, a systematic review and meta-analysis regarding the role of D-mannose in reducing UTI recurrence in adult women with recurrent UTIs compared with other prevention agents. The comparison of D-mannose with preventive antibiotics yielded a pooled relative risk for recurrent UTIs of 0.39 compared to 0.23 of the pooled relative risk of D-mannose only [25].

## 3. Chondroitin Sulphate

In recent years, chondroitin sulphate has emerged as a potential candidate for UTI prevention [26,27,28]. Chondroitin sulphate is a naturally occurring compound found in connective tissues, including those lining the urinary tract. The urothelium of the bladder is indeed coated by a thick layer of glycosaminoglycans (GAGs), which acts as a non-specific anti-adherence factor and defense mechanism against infections and irritants. Two types of GAGs are reported in bladder urothelium: non-sulphated GAGs as the hyaluronic acid and sulphated GAGs as heparan sulphate, chondroitin sulphate and dermatan sulphate. Chondroitin sulphate is known for its ability to maintain tissue integrity and provide structural support [29,30]. By supplementing with chondroitin sulphate, it is hypothesized that the protective effects on the urinary tract lining can help prevent the attachment and colonization of uropathogenic bacteria, reducing the risk of UTIs. Similar to D-mannose, chondroitin sulphate has been shown to interfere with bacterial adhesion [26]. Uropathogenic bacteria, such as *E. coli*, utilize adhesins to attach to the urinary tract epithelium [31]. Chondroitin sulphate can act as a decoy molecule, competing with these adhesins for binding sites and reducing bacterial attachment. This inhibition of bacterial adhesion can impede the initial steps of UTI development. Additionally, chondroitin sulphate has been found to have immunomodulatory properties, which can influence the immune response within the urinary tract. By regulating immune cell activity and cytokine production, chondroitin sulphate may contribute to a balanced immune response, helping to control inflammation and prevent the progression of UTIs [32]. Chondroitin sulphate supplementation has shown promise in reducing the frequency of UTI recurrences, both via intravesical and oral administration. Damiano et al. reported, in a prospective, randomized double-blinded placebo-controlled study including 57 women, a decreased UTI rate per patient per year (−86.6% versus −9.6%) and mean time of UTI recurrence (185.2 versus 52.7 days) in patients treated with intravesical instillation of hyaluronic acid plus chondroitin sulphate [33]. Similarly, Rahnnama’i et al., reported, in a retrospective study involving a total of 151 patients with recurrent UTI undergoing intravesical therapy with chondroitin sulphate only, a statistically significant reduction of the number of infections from 7.10 to 0.45 infection episodes per year as well as a reduction in the number of visits to the urologist from 7.46 to 1.28 episodes per year. Interestingly, these findings were comparable to patients treated with low-dose long-term antibiotics [28]. In a smaller study performed by De Vita et al. on 28 women followed for one year, the authors also demonstrated how intravesical instillation of hyaluronic acid plus chondroitin sulphate for 4 weeks and then once every 2 weeks, compared to long-term antibiotic prophylaxis using sulfamethoxazole and trimethoprim, reported similar results in terms of UTI recurrence, pain symptoms, sexual function, and quality of life [34]. Lastly, in regards to concerns surrounding the intravesical administration of chondroitin sulphate, an older meta-analysis involving four studies, for a total of 143 patients, reported a significant decrease in UTI rate per patient per year (−3.41, 95% confidence interval (CI) −4.33 to −2.49, *p* < 0.00001) as well as a longer mean UTI recurrence time (187.35 days, 95% CI 94.33–280.37, *p* < 0.0001) in patients treated with chondroitin sulphate compared to placebo [35]. More recently, Torella et al., reported in a prospective study involving 145 postmenopausal women, the efficacy of chondroitin sulphate, in combination with hyaluronic acid, curcumin, and quercetin, in the prevention of recurrent UTIs, with a significant improvement of related symptoms at a 12-month follow up [36]. Similarly, Schiavi et al. reported, in a study involving 98 consecutive patients of reproductive age affected by UTIs and treated with a combination of hyaluronic acid, chondroitin sulphate, curcumin, and quercetin administered orally, a significant decrease of dysuria episodes after 6 months of treatment. During the treatment, only 7.1% of patients experienced UTI recurrence, confirmed by a positive urine culture, proposing the oral combination of hyaluronic acid, chondroitin sulphate, curcumin, and quercetin as a valid and well-tolerated non-antibiotic treatment in the prevention of UTIs [37]. According to the previously reported findings, chondroitin sulphate may be a valuable addition to preventive strategies for recurrent UTIs. Additionally, combining chondroitin sulphate with other compounds, such as mannose and *N*-acetylcysteine, has been explored for their potential synergistic effects in UTI prevention [38,39]. The combination of chondroitin sulphate with other agents may therefore enhance the overall efficacy by targeting multiple steps in the pathogenesis of UTIs, including bacterial adhesion, biofilm formation, and immune modulation [40,41].

## 4. Hyaluronic Acid

Hyaluronic acid is a non-sulphated glycosaminoglycan and a major mucopolysaccharide widely found in connective, epithelial, and neural tissue, representing the main components of the extracellular matrix [42,43]. While its role in urinary tract infections (UTIs) is still being investigated, hyaluronic acid is thought to play a crucial role in maintaining the health and integrity of the urinary tract epithelium due to its protective properties, constituting a barrier against infections and irritants [44,45]. Similar to chondroitin sulphate, by supporting the health of the urinary tract epithelium and modulating the immune response, hyaluronic acid may contribute to reducing susceptibility to UTIs. Additionally, hyaluronic acid exhibits anti-inflammatory properties and could control the inflammatory cascade triggered by UTIs, reducing tissue damage and promoting healing. These characteristics, associated with the inhibition of adherence of immune complexes as well as cells, inhibition of leukocyte migration/aggregation, regulation of endothelial and fibroblast proliferation and, lastly, regulation of connective tissue healing, make this compound a key factor in the urothelial barrier [46,47,48]. Hyaluronic acid represents, indeed, a protective compound for the urothelial lining which, when it becomes permeable, allows the penetration of toxic substances and irritants into the bladder wall, producing a cascade of inflammatory response which results in additional pain and damage to the urothelium [49]. Clinical studies evaluating the efficacy of hyaluronic acid in UTIs are still limited, but initial findings are promising. Intravesical instillation of hyaluronic acid has been explored as a therapeutic approach in patients with recurrent UTIs and interstitial cystitis, reporting remarkable improvements in urinary symptoms, decreased UTI recurrence rates, and enhanced quality of life in treated individuals. One of the first studies to evaluate the efficacy of hyaluronic acid against recurrent UTIs was performed by Lipovac et al. on 20 women with a history of UTIs treated with nine intravesical instillations of hyaluronic acid over 6 months. The total number of UTIs were, before and after treatment, 67 and 10, respectively (*p* < 0.001), with 13 patients free of recurrences until the end of the study (mean follow of up 47.6 weeks). In the remaining seven women who had UTI recurrences, the mean time to recurrence was increased from 76.7 days to 178.3 days for a mean number of infections per year reduced from 4.99 to 0.56 (*p* < 0.001) [50]. Similar studies were conducted by Damiano et al., De Vita et al., and Torella et al., which involved the use of intravesical hyaluronic acid plus chondroitin sulphate and were already previously described [33,35,36]. In 2019, Scarneciu et al., conducted a clinical trial on 30 patients with UTIs, evaluating the efficacy of intravesical instillation of hyaluronic acid in reducing and relieving lower urinary tract irritation symptoms. A significant improvement in urinary bladder pain, day-time urinary frequency, and quality of life, assessed via questionnaires, was reported, in a mean follow-up of 15 months [51]. Lastly, a systematic review and meta-analysis by Goddard and Janssen related to the use of intravesical hyaluronic acid and chondroitin sulphate for recurrent UTIs reported, in a total of eight studies (two randomized and six non-randomized), a decreased UTI rate per patient per year, with a pooled mean difference of −2.56 (95% confidence interval [CI] –3.86, −1.26; *p* < 0.001) and an increased time to first UTI recurrence, with a pooled mean difference of 130.05 days (95% CI 5.84, 254.26; *p* = 0.04) [52]. While the evidence supporting the role of hyaluronic acid in UTIs is growing, more research is needed to establish standardized treatment protocols, determine optimal dosing regimens, and evaluate long-term safety. Additionally, the specific formulations and delivery methods of hyaluronic acid need to be considered to ensure efficient delivery and sustained effects in the urinary tract. Indeed, it has to be considered that oral hyaluronic acid supplementation, which could further improve the compliance of treatment, has to be evaluated in terms of bioavailability and absorption considering that the size of the molecule and its breakdown during digestion which could affect its therapeutic potential [53,54].

## 5. *N*-acetylcysteine

NAC has gained recognition for its potential role in the management of UTIs, particularly in the context of recurrent UTIs. NAC is a precursor of the antioxidant glutathione and possesses several mechanisms that make it a promising adjunctive therapy for recurrent UTIs. In particular, NAC acts as a potent antibiofilm agent both alone and in combination with antibiotics against *E. coli*, *Enterococcus faecalis*, and other species associated with the production of bacterial biofilms [46,47,48]. Additionally, it seems that NAC penetrates the biofilm matrix and it is able to kill the bacteria within, via the dissociation and acidification of bacterial cytoplasm, leaving an empty gel-like matrix [55]. A concentration of 2 g per liter of NAC is able to inhibit *E. coli* biofilm formation by 20–40%, rising to 90% at a concentration of 4 g per liter [56,57]. The disruption of the bacterial biofilm, which represents a protective mechanism used by bacteria to resist the host immune response and antibiotic treatment, enhances the susceptibility of bacteria to conventional antibiotics, making them more effective in eradicating the infection [58,59]. A study by Manoharan et al. performed on bladder epithelial cells involved the use of bacteria which were added to the cell culture in the presence of NAC, allowing the bacterial invasion. NAC completely inhibited the invasion of bladder epithelial cells both in the presence of *E. coli* and *E. faecalis*, in a dose-dependent manner, displaying no cytotoxicity against the cells. Additionally, NAC damaged bacterial membranes and prevented biofilm formation. The effects were amplified when an antibiotic, in addition to NAC, was administered [60]. Other favorable effects of NAC are correlated to its immunomodulating activity as well as oxidative stress reduction, permitting an ability to maintain and improve the bladder mucosal protection. Although no studies have been conducted regarding the effects of NAC on bladder urothelial cells in terms of immunomodulation and reduction of oxidative stress, its effects on pulmonary and bronchial epithelium are well-known [61,62,63]. In particular, regarding concerns surrounding immunomodulation activity, UTIs involve complex interactions between the pathogen and the host immune system. The immunomodulatory properties of NAC could regulate the immune response in the urinary tract, enhancing the activity of immune cells, such as neutrophils and macrophages, which play crucial roles in eliminating bacteria [64,65]. Additionally, NAC can reduce the production of pro-inflammatory cytokines and promote an anti-inflammatory environment, thereby minimizing tissue damage and inflammation associated with UTIs [66,67]. The effects of NAC on oxidative stress reduction are, similarly, well-known. Considering that UTIs are characterized by increased oxidative stress in the urinary tract, which can contribute to tissue damage and exacerbate the inflammatory response, NAC could act as a potent antioxidant by replenishing intracellular glutathione levels and scavenging reactive oxygen species. The reduction of oxidative stress could therefore alleviate the damage caused by UTIs and support the healing process [68,69,70]. Lastly, the urinary tract’s mucosal lining acts as a physical barrier against bacterial colonization. Disruption of this barrier can increase the susceptibility to UTIs. NAC has been shown to enhance the integrity of the mucosal lining, thereby potentially reducing the adhesion of bacteria to the urinary tract epithelium. This protective effect could help prevent the initial attachment and colonization of uropathogenic bacteria, reducing the likelihood of recurrent UTIs [71,72]. All these effects of NAC, in combination with other agents such as mannose and chondroitin sulphate, could be furtherly enhanced, providing synergistic effects by targeting different aspects of UTI pathogenesis, including bacterial adhesion, biofilm formation, and immune modulation. Although, further studies are required in order to fully evaluate the role of NAC on the urothelial epithelium.

## 6. Cranberry

The role of cranberry (*Vaccinium macrocarpon*) in the prevention and management of UTIs has garnered substantial attention within the scientific community due to the unique composition of bioactive compounds, mostly proanthocyanidins, anthocyanidins, and flavonols, which have been shown to interfere with the adhesion of uropathogens to the urothelial cells, a crucial step in the initiation of infection [73,74]. In addition, cranberry consumption has been associated with a decrease in UTI-related symptoms via the suppression of inflammatory cascades [75]. Nevertheless, despite its historical association with urinary tract health, the mechanism implied in the preventive effects of cranberry consumption against UTIs is still not completely understood. It is however verified that in ex vivo studies, cranberry products have shown an antiadhesive activity as reported by Liu et al., which showed how the urine samples collected from healthy subjects consuming cranberry products reported antiadhesive properties compared to those collected from a placebo group [76]. Similarly, Baron et al. showed a significant reduction in the adherence and biofilm formation of Candida albicans strains in urine collected after the intake of cranberry products [77]. Concerning the effects of cranberry products in vivo, several recent studies have reported interesting results. Maki et al., in a randomized, double-blind, placebo-controlled multicenter clinical trial involving 373 women with a recent history of UTIs consuming 240 mL of cranberry beverage per day, reported a 40% reduction in the incidence of UTIs compared to the placebo with one clinical UTI event prevented for every 3.2 women [78]. In an analogous study by Koradia et al. involving 81 women followed up for 26 weeks, the supplementation of cranberry products significantly lowered the number of UTIs (with 90% of patients in the intervention group which did not report a UTI event compared to 67% of the control group) and increased the time to first UTI (174 days versus 90 days) [79]. Lastly, a meta-analysis by Fu et al., which considered seven randomized controlled trials for a total of 1498 healthy women, reported a reduced risk of UTIs by 26% in subjects consuming cranberry products. However, due to the limited size of the studies analyzed, with only two studies involving over 300 participants, the findings obtained should be confirmed by better and larger studies [80]. Another more recent meta-analysis by Xia et al., analyzing 23 trials for a total of 3979 participants, found that cranberry-based products could significantly reduce the incidence of UTIs in susceptible populations, especially in those assuming cranberry juice compared to capsules or tablets (with a relative risk reduction of 35%). Nevertheless, considering the limitations of the studies involved (insufficient information included, differences and inconsistencies in dosages and compositions of cranberry products, and limited sample sizes), the authors suggested a certain caution in the interpretation of the results [81]. Indeed, the inconsistency in meta-analysis methodologies and methodological heterogeneity could have led to varying results and interpretations, contributing to enriching the uncertainty regarding the efficacy of cranberry in UTI management and prophylaxis [82]. Also due to these reasons, other ongoing studies are currently investigating the role of cranberry in UTIs [83,84].

## 7. The Use of Combined Compounds in UTIs: A Synergistic Approach Targeting the Bladder

The combined use of previously described compounds permits targeting multiple aspects of UTI pathogenesis, combining the individual properties of every compound in a synergistic approach aimed to provide a comprehensive and multifaceted approach to UTI prevention and management. To summarize, mannose could interfere with bacterial adhesion to the urinary tract epithelium, reducing the initial step of UTI development; chondroitin sulphate and hyaluronic acid protect the urinary tract lining, inhibit bacterial adhesion, and modulate the immune response while NAC exhibits antimicrobial effects, modulates the immune response, reduces oxidative stress, and may have a role in recurrent UTI prevention [28,37,60,85]. Therefore, when used in combination, these compounds could act synergistically, targeting various stages of UTI pathogenesis. According to these premises, the role of an oral supplement such as Uroial PLUS^®^, which include all the previously reported compounds, could represent a novelty in the urological panorama due to the high concentrations of D-mannose (3000 mg), chondroitin sulphate (500 mg), NAC (600 mg), and hyaluronic acid (35 mg). By targeting multiple mechanisms, the combined approach may enhance the overall effectiveness of UTI prevention and management. It is important to note that the optimal dosages, treatment durations, and specific combinations of these compounds require further research and clinical investigation. Additionally, individual variations, underlying health conditions, and UTI risk factors should be considered when determining the most suitable combination therapy for each patient. Further compounds and nutraceuticals could be further added and integrated into a multifaceted approach to UTI [86].

## 8. The Use of Combined Compounds in UTIs: A Synergistic Approach Targeting the Gut

Recently, a new intestinal mucosal protective effect was described as a consequence of the interaction of two of these compounds, specifically cranberry and chondroitin sulphate in a specific mass ratio [26]. The precipitate formed can act as a barrier agent with a mucosal protective effect by improving the preservation of the physiological intestinal barrier and the tight junctions of epithelial cells thus reducing the risk of bacterial translocation from the gut to the urological tract. In particular, by forming a covering layer on the mucosa, the mucosal barrier prevents the adhesion of *E. coli* (mediated by the fimbriae) and consequently the invasion of the epithelium. In fact, in the face of excessive intestinal colonization by uropathogens (which by their nature tend to adhere), the presence of the protective film prevents their nesting at the epithelial level, favoring their elimination. This second part of the mechanism of action is as important as the first, as an accumulation of uropathogens in the intestine with the formation of a bacterial reservoir is an important risk factor for urinary colonization, according to the fecal-perineal-urethral hypothesis, according to which the proximity of the urethra to the terminal tract of the colon is responsible for the external migration of uropathogens and for the contamination of the urinary tract [87,88,89].

Furthermore, when cranberry and chondroitin sulphate are in combination with other compounds, specifically D-mannose, hyaluronic acid (HA), and NAC, the biological effects applied to UTI pathogens virulence showed a significant reduction of biofilm production, especially on antibiotic-resistant strains of uropathogens [26,90,91]. Based on these effects, new mucosal protective agents such as compounds are herein assessed to represent a new potential approach against recurrent cystitis and UTIs that originate from intestinal dysfunction (Table 1).

## 9. Conclusions

The role of D-mannose in UTIs is well-established and known, but other interesting and widely used compounds such as hyaluronic acid, chondroitin sulphate, and NAC, are starting to quickly deliver interesting findings considering that their combination may represent a novel therapeutic approach. In fact, the combination of these compounds targets both the bladder and the gut, offering a novel mechanism of action for the prevention and management of UTIs. The combined approach, i.e., the use of multiple compounds synergically, could offer an even more comprehensive and effective approach to urinary health and UTI prevention. Future research should focus on well-designed clinical trials to establish the safety, efficacy, and optimal combinations of these compounds, alone and in combination, additionally understanding the potential interactions and compatibility of the combined compounds to provide the best therapeutic effectiveness.

## Figures and Tables

**Table 1:** Main effects of compounds reported on the urothelial barrier.

Compound	Main Effect on Urothelial Barrier
D-mannose	Inhibits bacteria adhesion [17,92]
Chondroitin sulphate	Maintain urothelium impermeability and tissue integrity [30,93]
Hyaluronic acid	Maintain urothelium impermeability, modulate the immune response, regulate tissue healing [44,45]
*N*-acetylcysteine	Antioxidant activity, impedes bacterial biofilm, reduction of oxidative stress [56,92,94]
Cranberry	Inhibits bacteria adhesion, potential reduction of inflammation and oxidative stress [73,74]

## Data Availability

No new data were created or analyzed in this study. Data sharing is not applicable to this article.
